# Pan-cancer analysis of m^5^C regulator genes reveals consistent epigenetic landscape changes in multiple cancers

**DOI:** 10.1186/s12957-021-02342-y

**Published:** 2021-07-29

**Authors:** Yuting He, Xiao Yu, Menggang Zhang, Wenzhi Guo

**Affiliations:** 1 Department of Hepatobiliary and Pancreatic Surgery, The First Affiliated Hospital of Zhengzhou University, No.1 Jianshe Road, Zhengzhou, 450052 China; 2 Key Laboratory of Hepatobiliary and Pancreatic Surgery and Digestive Organ Transplantation of Henan Province, The First Affiliated Hospital of Zhengzhou University, Zhengzhou, 450052 China; 3 Open and Key Laboratory of Hepatobiliary & Pancreatic Surgery and Digestive Organ Transplantation At Henan Universities, Zhengzhou, 450052 China; 4 Henan Key Laboratory of Digestive Organ Transplantation, Zhengzhou, 450052 China

**Keywords:** m^5^C regulatory genes, Frequent network mining, Pan-cancer analysis, Survival, 5-Methylcytosine

## Abstract

**Background:**

5-Methylcytosine (m^5^C) is a reversible modification to both DNA and various cellular RNAs. However, its roles in developing human cancers are poorly understood, including the effects of mutant m^5^C regulators and the outcomes of modified nucleobases in RNAs.

**Methods:**

Based on The Cancer Genome Atlas (TCGA) database, we uncovered that mutations and copy number variations (CNVs) of m^5^C regulatory genes were significantly correlated across many cancer types. We then assessed the correlation between the expression of individual m^5^C regulators and the activity of related hallmark pathways of cancers.

**Results:**

After validating m^5^C regulators’ expression based on their contributions to cancer development and progression, we observed their upregulation within tumor-specific processes. Notably, our research connected aberrant alterations to m^5^C regulatory genes with poor clinical outcomes among various tumors that may drive cancer pathogenesis and/or survival.

**Conclusion:**

Our results offered strong evidence and clinical implications for the involvement of m^5^C regulators.

**Supplementary Information:**

The online version contains supplementary material available at 10.1186/s12957-021-02342-y.

## Background

Cancers have become the second life-threatening malignancies, which contribute to almost 18.1 million people occurred and 9.6 million death globally in 2018 [1]. Lack of efficient diagnosis indicators at an early stage and high rate of postoperative recurrence contribute to poor clinical prognosis and high mortality [2, 3]. Growing evidence demonstrated that genomic instability [4, 5], oncogene activation, aberrant methylation modifications, alterations in epigenetic changes [6–8], aberrant expression of microRNAs [9], and alterations of signaling pathways are crucial factors and contribute to cancer pathogenesis [10–12]. Methylation is an essential epigenetic modification and is closely related to the pathogenesis of cancers [13–17]. The 5-methylcytosine (m^5^C), N6-methyladenine (m^6^A), and N1-methyladenosine (m^1^A) have become the most common types of epigenetic modifications in eukaryotes [13]. Emerging evidence has demonstrated that m^5^C modification has the potential to serve as novel epigenetic markers with remarkable biological significance in biological processes [18–20].

m^5^C modification distributes in different types of RNAs and DNAs [21–23]. m^5^C modifications can even modify the destiny of cancer cells [24]. m^5^C regulators contain writers, erasers, and readers, which function as common epigenetic modification and contribute to pre-mRNA splicing, gene expression, gene silencing, nuclear export, genomic maintenance, and translation initiation modifications [25, 26]. m^5^C could therefore be used as a biomarker for disease progression, including various types of cancers [27]. m^5^C maintains open and closed chromatin states to control gene expression, genome editing, organismal development, and cellular differentiation [23]. In this context, writers act within a methyltransferase complex to methylate targets, and erasers remove m^5^C methylation, while readers recognize and bind to m^5^C-methylated RNA and implement corresponding functions [25, 28, 29]. The anomalous interplay between writers and erasers, arising from alterations to their expression, has been linked to cancer pathogenesis and progression [23, 30]. However, pan-cancer effects of changes to m^5^C regulatory gene expression have not been fully defined. Next-generation sequencing (NGS) provides us effective tools to comprehensively view the m^5^C distribution landscape throughout the global transcriptome [27].

In this study, we identified the potential prognostic value of m^5^C regulators and provided a comprehensive understanding of m^5^C modifications in pan-cancers, which will help to find novel opportunities for cancer early detection, treatment, and prevention.

## Materials and methods

### Study workflow

**Fig. 1:**
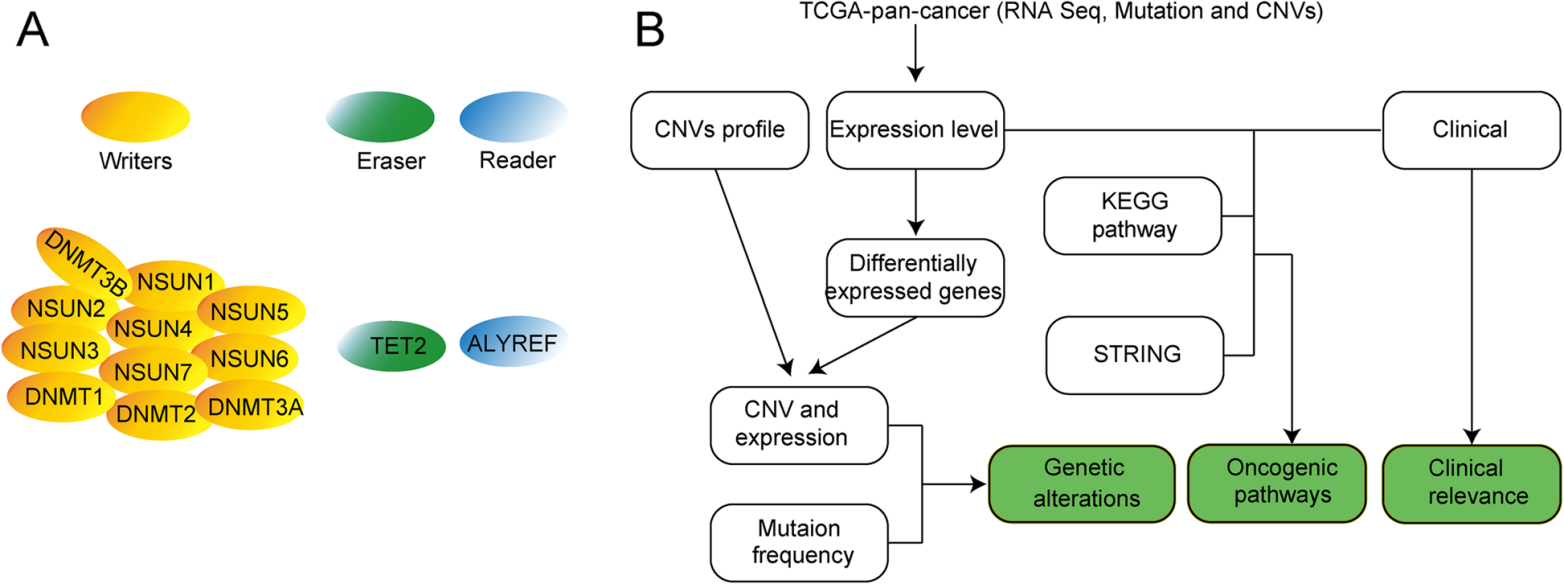
m^5^C regulators and the function in cancers. **A**. The distribution and the function of m^5^C regulatory writers, eraser, and reader. **B** The workflow scheme for this study

We downloaded fragments of kilobase transcripts based on fragments per kilobase of transcript per million (FPKM) gene expression from The Cancer Genome Atlas (TCGA, https://www.cancer.gov/) dataset among 33 different cancer types. m^5^C regulator patterns were investigated in 5480 samples among 33 different cancer types, including somatic mutations, copy number variations (CNVs), gene expression, and RNA-seq data (Fig. 1B).

### Genomic data collection of m^5^C regulators

Thirteen m^5^C regulators were identified from published papers. Information of m^5^C regulators was collected from Gene Cards (www.genecards.org). Ensemble gene IDs and HUGO Gene Nomenclature Committee symbols were assigned to each m^5^C regulator-associated gene.

### Whole genomics data analysis of 33 pan-cancers

The integrated OMICS datasets based on the TCGA database of 33 pan-cancers were applied in this study. We collected the mutation annotation format profile from TCGA, which contains over 10,000 cancer patient’s information. The level 3 data of copy number alterations profiles in TCGA was acquired for secondary analysis. In addition, we downloaded pan-cancers RNAseq data of genomic variations profiles and corresponding clinical information from the Genomic Data Commons Data Portal using the R package “TCGAbiolinks.”

### Differently expression genes (DEGs) identification

The DESeq package in R language was utilized to validate the DEGs between 33 pan-cancer samples and adjacent samples. Genes with a mean value > 0 were included in the screening of DEGs. To establish proper DEGs among 33 cancers, we settled the adjusted *P* value less than 0.01 and |log2 fold change (log2Fc) | no less than two as the statistical threshold value for differentially expressed genes. The results were screened as significant DEGs and methylated sites.

### Functional annotations and pathway enrichment analysis

To evaluate the biological functions of each m^5^C modification-related gene, we transformed the RNA-seq data of all samples into transcripts per million (TPM) values. The methods have been described in a previous study [31]. The insufficient, duplicated, and zero expression genes will be eliminated. Furthermore, the gene set variation analysis (GSVA) was applied to determine transcriptomic activities and explore the biological processes of m^5^C regulators. To further explore m^5^C regulators related inhibition and activation factors, we performed the Pearson correlation coefficient (PCC) and defined the absolute value of the PCC greater than 0.5 and *p* value of less than 0.01 as the screen cut-off. The results could be recognized as significantly correlated m^5^C regulators.

### The internships between m^5^C regulators

To visualize the intercorrelations among m^5^C regulators, we adopted the “CORPRRAP” R package (https://github.com/taiyun/corrplot). Besides, the STRING database was also applied for the exploration and analysis of these associations between m^5^C regulators and 325 related genes [32]. The 325 genes were obtained from the Kyoto Encyclopedia of Genes and Genomes (KEGG) database (http://www.kegg.jp/ or http://www.genome.jp/kegg/). The correlations between m^5^C regulators and 325 genes were visualized through Cytoscape (https://cytoscape.org/).

### Clinical characteristic of m^5^C regulators

To explore the m^5^C regulators’ related clinical characteristics, we classified genes into high and low expression groups based on genes’ median expression. Correlations between outcomes of the two groups were then analyzed through a log-rank test via R software (https://cran.r-project.org/web/packages/survival/index.html). The log-rank test was performed to weigh the overall survival rates that differ between the high and low expression groups. The CRAN Package survival (https://cran.r-project.org/web/packages/survival/index.htm) was performed, and we defined the *p* value of less than 0.05 as significant difference.

### The roles of m^5^C regulators in cell growth

The CRISPR-CAS9 gene scale screening of cell lines from 33 cancer types was collected from previous study [32]. We calculated the proportion of every regulator as an essential gene in the cell lines.

## Results

### Results m5C regulators identification and its genomic extensive genetic changes

**Fig. 2:**
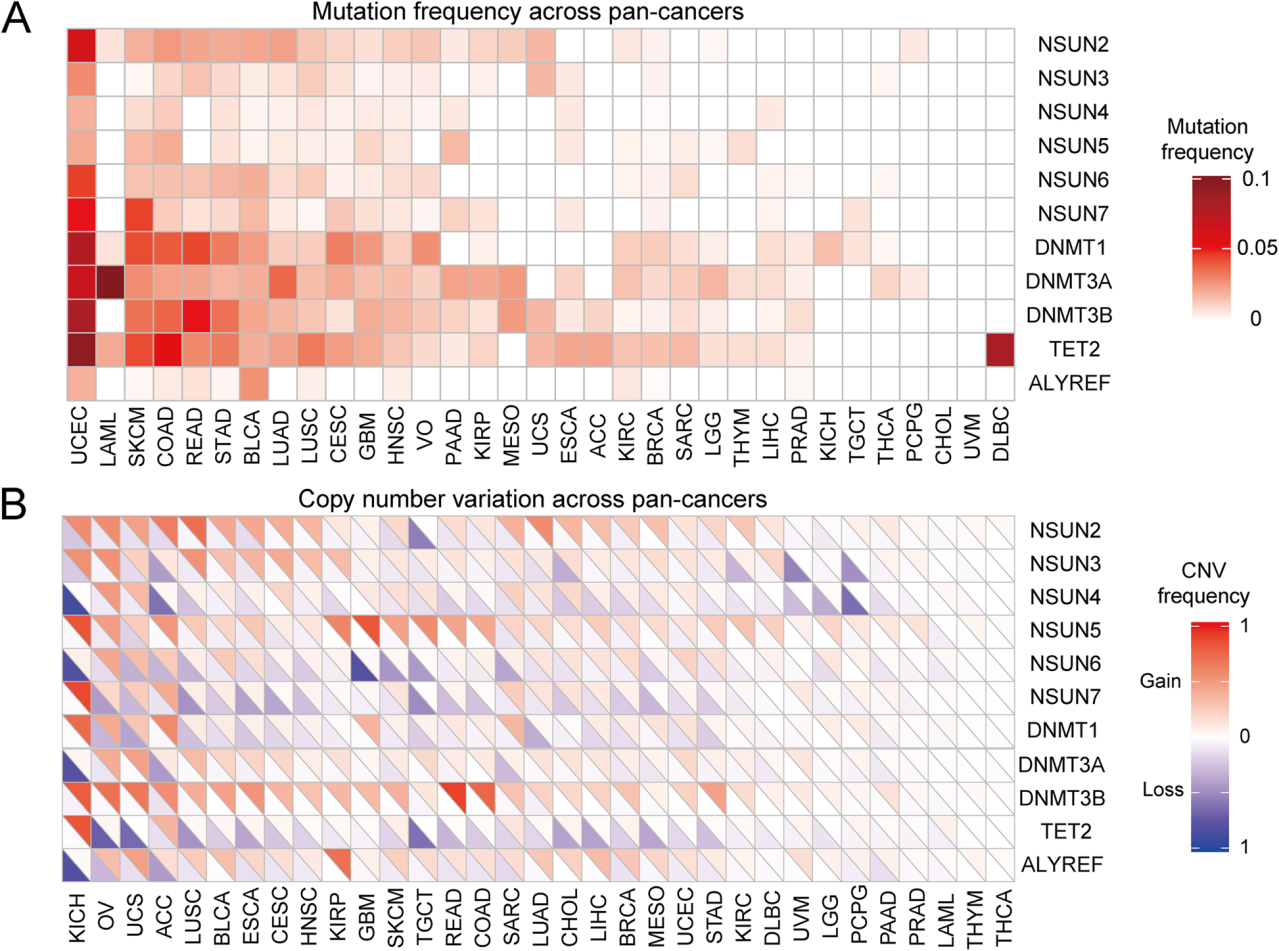
Mutation and CNV of m^5^C regulators across pan-cancer. **A** The mutant frequency of m^5^C regulators across 33 cancer types. **B** CNV analysis of m^5^C regulators across cancer types. The upper part of each grid shows the deletion frequency, and the bottom part shows the amplification frequency

In this study, we identified 13 m^5^C regulators, as shown in Fig. 1A, eleven writers (NSUN1-7, DNMT1, DNMT2, DNMT3A, and DNMT3B), one eraser (TET2), and one reader (ALYREF). This study validated the frequency of m^5^C regulator patterns among 33 cancers by integrating somatic mutations and CNVs data. Table 1 illustrated detailed information. The results indicated that the overall average mutation frequency of regulatory factors is low, ranging from 0 to 9% (Fig. 2A). The uterine corpus endometrial carcinoma (UCEC) is characterized as a high tumor mutation burden [33]. The UCEC showed significantly higher mutation frequency. Horizontal analysis indicated that TET2, DNMT3B, DNMT3A, and DNMT1 demonstrated much higher mutation frequency among 33 cancers. Furthermore, we uncovered the CNV mutation frequency of m^5^C regulators was common. Regulators such as DNMT3B, ALYREF, and NSUN5 displayed extensive CNVs. On the contrary, TET2 and NSUN4 showed significant lack of m^5^C modification related CNV mutations among pan-cancers (Fig. 2B, and Table 2).

**Table 1:** The 33 cancer types in TCGA pan-cancer project

Cancer types	Abbr	Normal tissues	Cancer tissues	Mutation	CNV
Kidney Renal Clear Cell Carcinoma	KIRC	72	539	370	531
Kidney Renal Papillary Cell Carcinoma	KIRP	32	289	282	291
Kidney Chromophobe	KICH	24	65	66	69
Brain Lower Grade Glioma	LGG	0	529	526	516
Glioblastoma Multiforme	GBM	5	169	403	580
Breast Invasive Carcinoma	BRCA	113	1109	1026	1083
Lung Squamous Cell Carcinoma	LUSC	49	502	485	504
Lung Adenocarcinoma	LUAD	59	535	569	519
Rectum Adenocarcinoma	READ	10	167	151	168
Colon Adenocarcinoma	COAD	41	480	408	454
Uterine Carcinosarcoma	UCS	0	56	57	59
Uterine Corpus Endometrial Carcinoma	UCEC	35	552	531	542
Ovarian Serous Cystadenocarcinoma	OV	0	379	412	582
Head and Neck Squamous Carcinoma	HNSC	44	502	509	525
Thyroid Carcinoma	THCA	58	510	500	502
Prostate Adenocarcinoma	PRAD	52	499	498	495
Stomach Adenocarcinoma	STAD	32	375	439	444
Skin Cutaneous Melanoma	SKCM	1	471	468	370
Bladder Urothelial Carcinoma	BLCA	19	414	411	411
Liver Hepatocellular Carcinoma	LIHC	50	374	365	373
Cervical Squamous Cell Carcinoma and Endocervical Adenocarcinoma	CESC	3	306	291	298
Adrenocortical Carcinoma	ACC	0	79	92	93
Pheochromocytoma and Paraganglioma	PCPG	3	183	184	165
Sarcoma	SARC	2	263	239	260
Acute Myeloid Leukemia	LAML	0	151	141	194
Pancreatic Adenocarcinoma	PAAD	4	178	178	187
Esophageal Carcinoma	ESCA	11	162	185	187
Testicular Germ Cell Tumors	TGCT	0	156	151	153
Thymoma	THYM	2	119	123	126
Mesothelioma	MESO	0	86	82	90
Uveal Melanoma	UVM	0	80	80	83
Lymphoid Neoplasm Diffuse Large B-cell Lymphoma	DLBC	0	48	37	51
Cholangiocarcinoma	CHOL	9	36	36	39
In total		730	10,363	10,295	10,944

**Table 2:** The mutation frequency of m5C regulators across 33 cancer types (Top 5)

Function	Genes	UCEC	SKCM	COAD	READ	STAD
writers	NSUN1	0	0	0	0	0
	NSUN2	0.0622642	0.0192719	0.0250627	0.0218978	0.020595
	NSUN3	0.0283019	0.0021413	0.0100251	0.0145985	0.0091533
	NSUN4	0.0188679	0.0085653	0.0125313	0	0.006865
	NSUN5	0.0207547	0.0171306	0.0200501	0	0.006865
	NSUN6	0.045283	0.0149893	0.0150376	0.0145985	0.0183066
	NSUN7	0.0509434	0.0449679	0.0125313	0.0072993	0.0091533
	DNMT1	0.0773585	0.0428266	0.0401003	0.0437956	0.0320366
	DNMT2	0	0	0	0	0
	DNMT3A	0.0660377	0.0278373	0.0225564	0.0218978	0.0183066
	DNMT3B	0.0811321	0.0342612	0.037594	0.0510949	0.0343249
eraser	TET2	0.0943396	0.0428266	0.0551378	0.0291971	0.0320366
reader	ALYREF	0.0188679	0.0021413	0.0050125	0.0072993	0.0022883

**Fig. 3:**
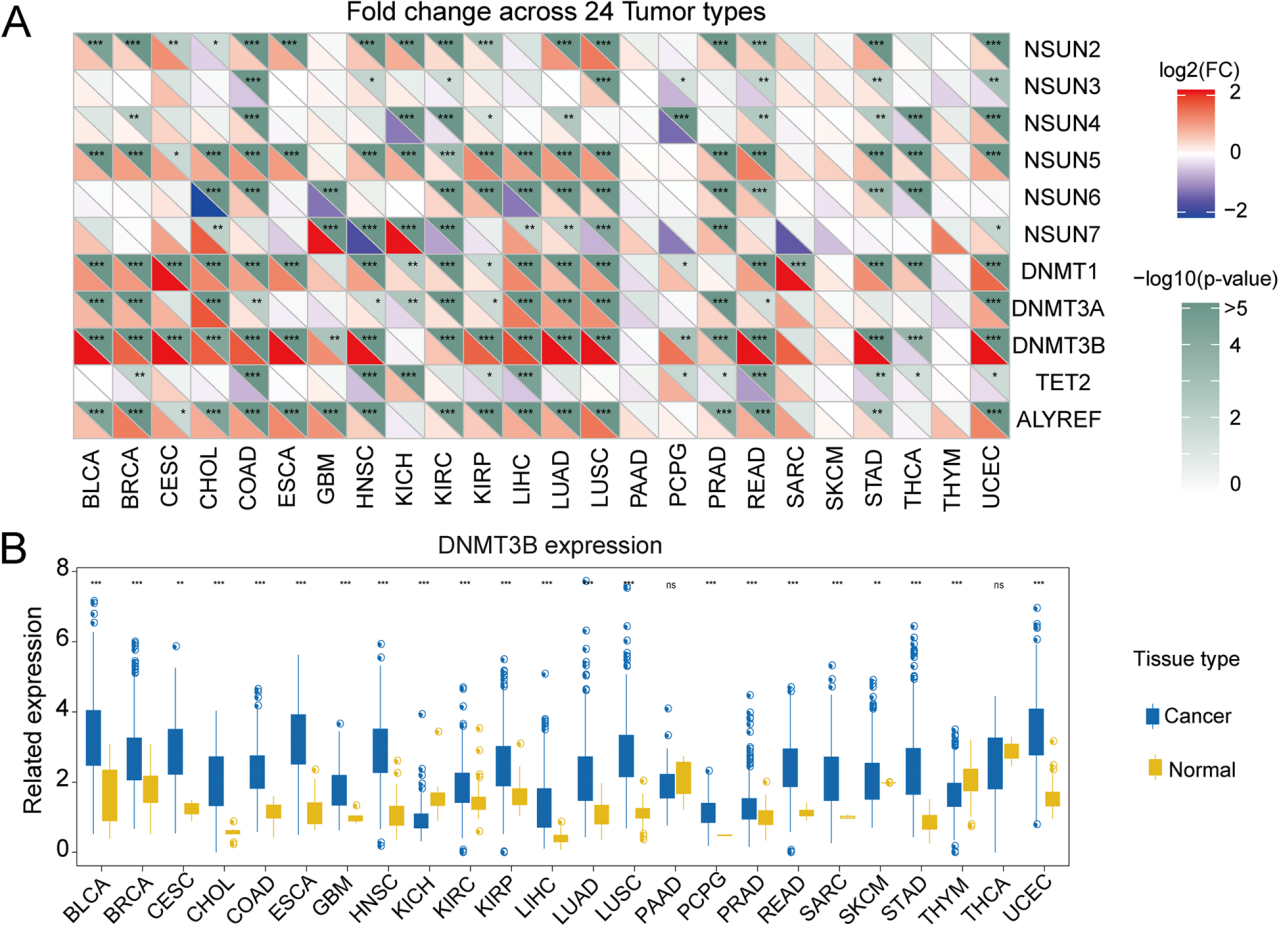
The association between CNV and the gene expression of m^5^C regulatory genes. **A** Alterations to m^5^C regulatory gene expression in 24 cancer types. The heat map demonstrates fold change, with red representing upregulated genes and blue representing downregulated genes. **B** Box plots exhibit the expression distribution of DNMT3B across tumor and normal samples in 24 cancer types

To further investigate whether the genomic mutations affect m^5^C regulators expression, we intensively detected the m^5^C regulators’ gene expression disturbances in thirty-three pan-cancers and five standard control samples. The result implied that the CNV alterations (amplification and deletion) might profoundly affect the m^5^C regulator’s expression (Fig. 3A). The m^5^C regulators with CNV amplification showed significantly increased expression in pan-cancers (such as DNMT3B), and m^5^C regulators with CNV deletion exhibited remarkably decreased expression, like TET2. In addition, we comparatively analyzed the m^5^C regulators’ expression levels in cancers and corresponding normal tissues and found out that DNMT3B was significantly overexpressed in thirty-three tumor or cancer tissues compared with adjacent normal tissues (Fig. 3B). These results uncovered that the m^5^C regulators among various cancers showed significant heterogeneity in gene expression and genetics. Collectively, our results demonstrated that aberrant m^5^C regulations were crucial for carcinogenesis and progression, which provided a clue for further functional detection.

### m^5^C regulators related pan-carcinogenic pathways

**Fig. 4:**
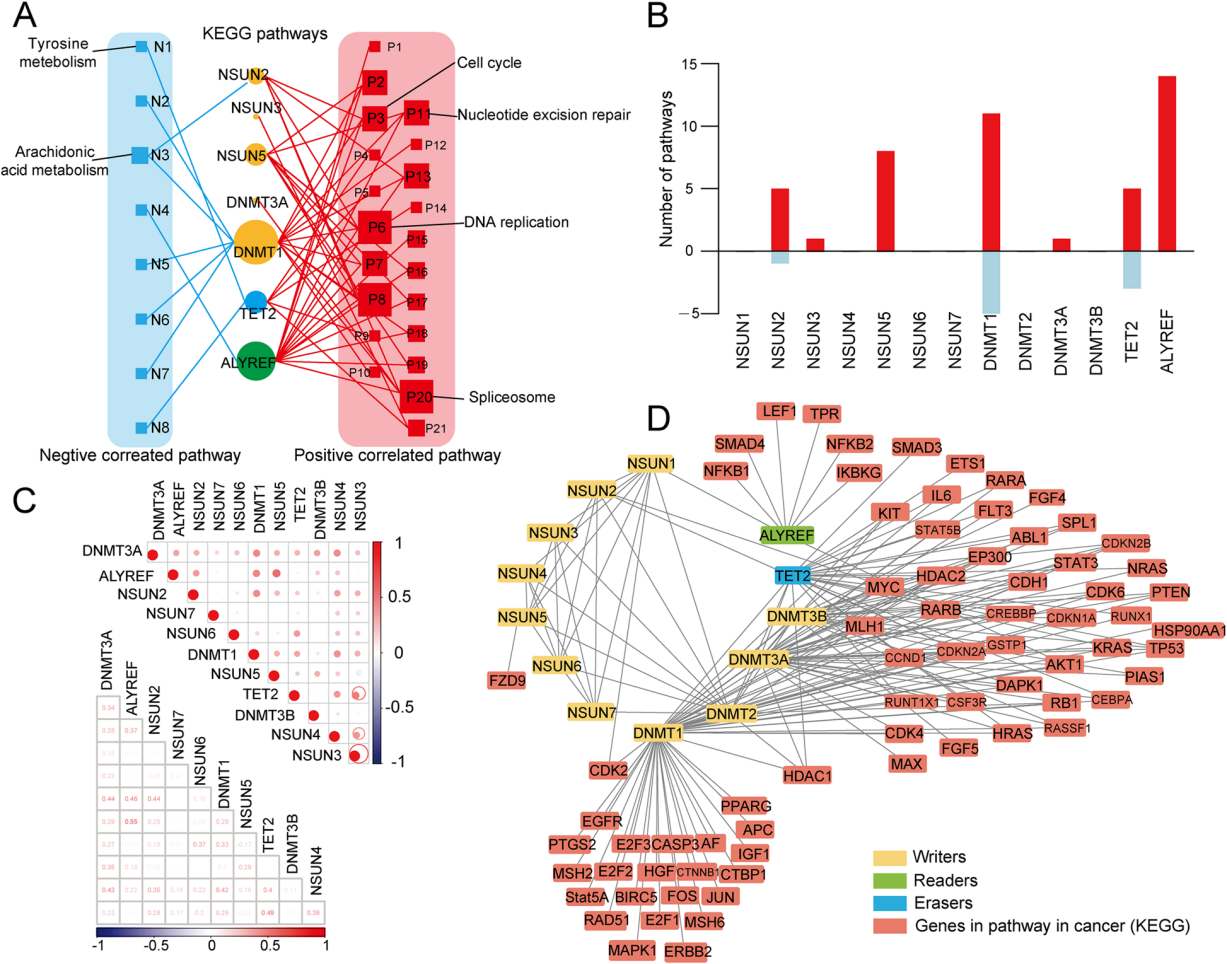
m^5^C regulators are associated with the activation and inhibition of cancer pathways. **A** Network landscape demonstrating the correlation between m^5^C regulators and cancer pathways. Red represents a positive correlation, and blue represents a negative correlation. The size of the nodes corresponds to the number of links. **B** The number of pathways correlated with individual m^5^C regulators. The upper panel represents positively correlated pathways, and the bottom panel represents negatively correlated pathways. **C** The correlation among the expression of m^5^C regulators. **D** The PPI network of m^5^C regulators

To comprehensively explore the molecular mechanism m^5^C regulators involved in cancers, we evaluated the correlations between m^5^C regulators’ proteins expression and KEGG enrichment analysis-related activities. The results indicated that m^5^C regulator proteins have a close relationship with tumor-related pathways’ activation and inactivation (Fig. 4A, and Table 3). ALYREF, DNMT1, and TET2 were involved in the cell cycle, DNA replication, and prostate cancer-related pathways. Notably, ALYREF was involved in multiple pathways, including cell cycle, DNA replication, and prostate cancer-related pathways. DNMT1 was involved in drug metabolism, lipid metabolism, and nucleic acid biosynthesis signaling pathways [34]. ALYREF, DNMT1, NSUN5, NSUN1, and TET2 showed active involvement in KEGG enrichment pathways (Fig. 4B). In addition, genes will not function alone [35]. Growing evidence indicated that genes always co-effect with multiple genes and always have multiple functions [35, 36]. We further explored the internal connections between m^5^C regulators gene expression. Results indicated that the readers, writers, and erasers also have high correlations with each other. The eraser TET2 was significant correlated with the writer NSUN3. Writers such as ALYREF and NSUN5 also showed obvious correlations (*R* = 0.55, *P* < 0.01) (Fig. 4C). Additionally, to visualize the interactions between m^5^C regulators, we utilized the protein–protein interaction (PPI) analysis in m^5^C regulators related proteins. The results showed that writers, readers, and erase were particularly frequent (Fig. 4D). These results indicated that interactions among m^5^C regulators play crucial roles in the development and progression of cancers.

**Table 3:** The CNV-Gain and CNV-loss frequency of m5C regulators across 33 cancer types (Top 5)

Genes	CNV Gain					CNV loss				
	KICH	OV	ACC	UCS	LUSC	KICH	ACC	TGCT	UCS	OV
NSUN1	0	0	0	0	0	0	0	0	0	0
NSUN2	0.560606	0.558678	0.633333	0.464286	0.699801	0.227273	0.144444	0.544872	0.125	0.099174
NSUN3	0.545455	0.540496	0.122222	0.232143	0.526839	0.212121	0.411111	0.102564	0.142857	0.044628
NSUN4	0.015152	0.499174	0.033333	0.339286	0.073559	0.863636	0.6	0.128205	0.089286	0.087603
NSUN5	0.818182	0.477686	0.511111	0.25	0.26839	0.030303	0.022222	0.032051	0.178571	0.082645
NSUN6	0.075758	0.428099	0.255556	0.321429	0.089463	0.787879	0.3	0.416667	0.321429	0.135537
NSUN7	0.863636	0.195041	0.411111	0.25	0.083499	0.015152	0.111111	0.49359	0.285714	0.408264
DNMT1	0.727273	0.418182	0.577778	0.285714	0.101392	0.015152	0.033333	0.198718	0.375	0.295868
DNMT2	0	0	0	0	0	0	0	0	0	0
DNMT3A	0.045455	0.403306	0.111111	0.464286	0.335984	0.772727	0.422222	0.012821	0.017857	0.102479
DNMT3B	0.787879	0.689256	0.555556	0.660714	0.39165	0.045455	0.111111	0.038462	0.017857	0.019835
TET2	0.818182	0.044628	0.366667	0	0.037773	0.015152	0.122222	0.608974	0.642857	0.694215
ALYREF	0.030303	0.320661	0.166667	0.464286	0.252485	0.787879	0.411111	0.038462	0.125	0.292562

### Clinical significance of m^5^C regulators in pan cancers

**Fig. 5:**
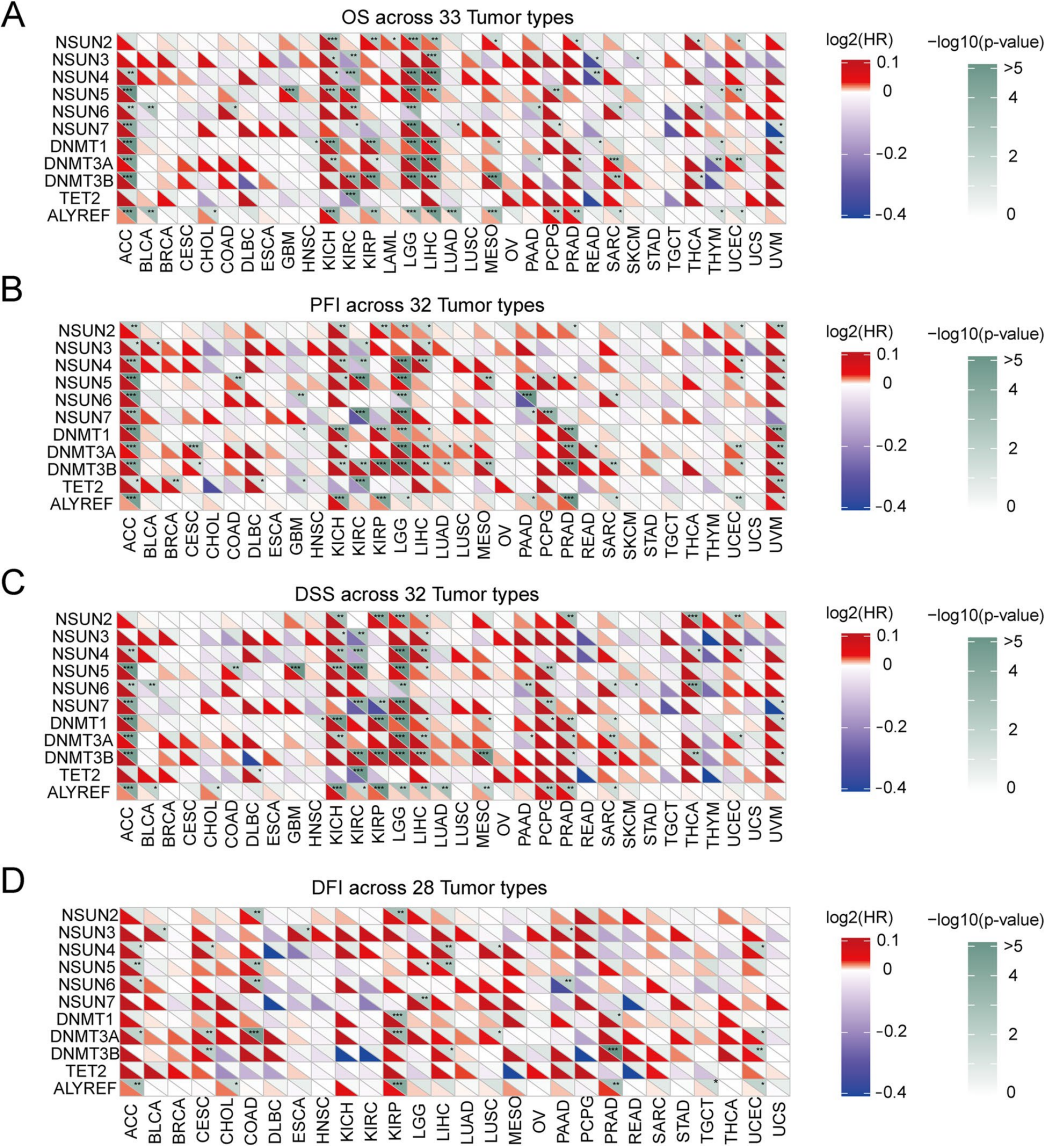
Summary of the relationship between m^5^C regulators expression and patient’s survival. **A** Overall survival (OS) of m^5^C regulators across 33 cancer types. **B** Progression-free interval (PFI) of m^5^C regulators across 32 solid cancer types. **C** Disease-specific survival (DSS) of m^5^C regulators across 32 solid cancer types. **D** Disease-free interval (DFI) of m^5^C regulators across 28 solid cancer types. Red represents a higher m^5^C regulator expression associated with poor survival, and blue represents an association with better survival

To evaluate the clinical prognosis of m^5^C regulators, we calculated the overall survival (OS), overall median progression-free interval (PFI), disease-specific survival (DSS), and disease-free interval (DFI) of m^5^C regulators. The OS analysis implied a significant correlation between m^5^C regulators and thirty-three pan-cancers. The heat map demonstrated that m^5^C regulators were significantly correlated with survival of patients, including OS, PFI, DSS, and DFI. In detail, the OS in adrenocortical carcinoma (ACC), kidney chromophobe (KICH), kidney renal clear cell carcinoma (KIRC), brain lower grade glioma (LGG), and liver hepatocellular carcinoma (LIHC) showed significant correlations with m^5^C regulators (Fig. 5A). The highly expressed DNMT3A, DNMT3B, DNMT1, and ALYREF were significantly related to poor prognosis. Collectively, the DNMT3A, DNMT3B, DNMT1, and ALYREF might function as poor prognosis predictors in cancer progression. Moreover, we evaluated the prediction of PFI at fixed time points in patients with thirty-two solid tumors. PFIs of DNMT3B and DNMT1 showed significantly higher hazard ratio values, indicating that they have the potential to be utilized as unfavorable prognosis prediction factors. The PFI in ACC, KICH, KIRC, LGG, and uveal melanoma (UVM) showed significant correlation ships with most m^5^C regulators (Fig. 5B). Similarly, The DSS and DFI analyses indicated that ACC and LGG showed remarkably correlations with most m^5^C regulators (Fig. 5C, D). These results indicated that m^5^C regulators have crucial prognostic prediction values in a variety of cancer types.

### Effect of m^5^C regulators in LIHC and cholangiocarcinoma (CHOL)

**Fig. 6:**
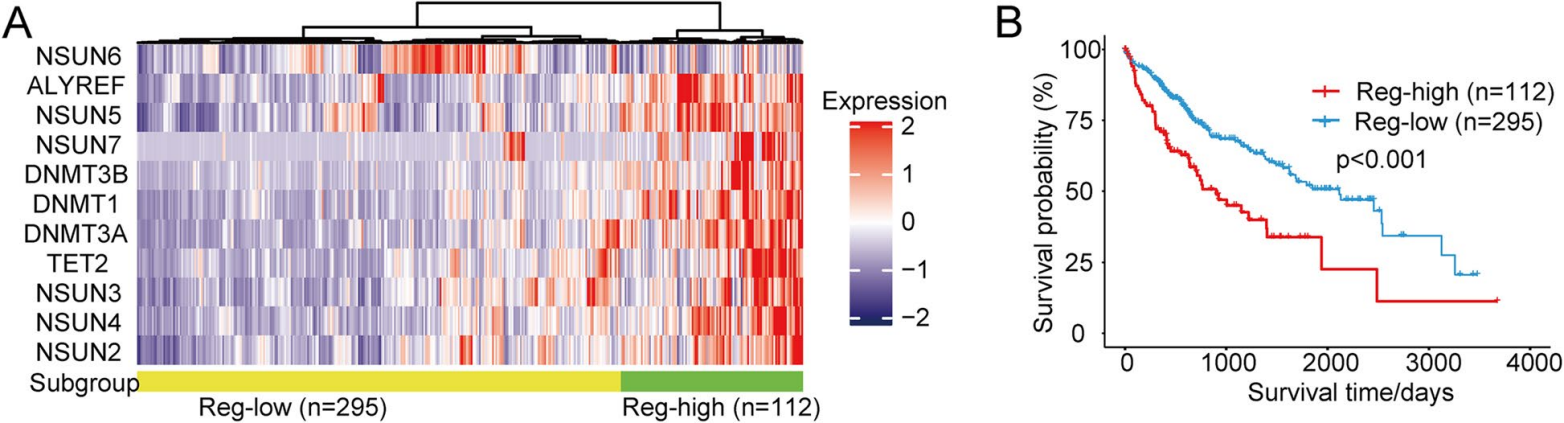
Effect of m^5^C regulators on patients with hepatocellular carcinoma and cholangiocarcinoma. **A** Heat map showing clustering for CHOL and LIHC patients based m^5^C regulator expression. Yellow represents Reg-low subgroup (*N* = 295), and green represents Reg-high subgroup (*N* = 112). **B** Kaplan–Meier survival plot of patients grouped by global m^5^C regulator expression pattern (*P* < 0.001)

Studies have validated that m^5^C-related genes alterations have a close relationship with advanced tumor progression and advanced tumor stages [37], based on the above pieces of evidence that most m5C regulators are associated with patients’ OS in LIHC and CHOL (Fig. 5A). Based on the overall expression patterns of m^5^C regulators, all patients in these cancer groups were categorized into two subgroups. The first subgroup consisted of 112 patients indicating high expression of m^5^C regulators (Reg-high), and the second subgroup consisted of 295 patients with low m^5^C regulators expression (Reg-low) (Fig. 6A). Compared with the Reg-high subgroup, the survival probability of patients in the Reg-low subgroup was significantly better (*P* < 0.001) (Fig. 6B). These results indicated that m^5^C regulators have a potential function as prognostic indicators in hepatocellular carcinoma and cholangiocarcinoma.

## Discussion

Epigenetic variation is often related to human disease, especially cancers [12, 38–40]. Remarkably progression has been made of various epigenetic-targeted therapies that have a broad application of malignancies and have exhibited detection and therapeutic potential for solid tumors in preclinical and clinical trials [41–45]. Aberrant methylation regulators process both in DNA and RNA play a critical role in epigenetic regulators, which are significantly associated with tumorigenesis [17, 46–51].

Original reports described that NSUN2 participates in catalyzing biological reactions of m^5^C formation in RNAs and regulating cell cycle [52], linked to stem cell differentiation and involved in progression [53]. It is also reported that m^5^C regulators such as NSUN2 and binding partner ALYREF participant in promoting mRNA export coordinately [54], and NSUN6, in complex with a full-length tRNA substrate targeting cytosine accessible to the enzyme for methylation [14, 55, 56]. The NSUN3 is required for the deposition of m^5^C at the anticodon loop in the mitochondria encoded transfer RNA methionine [57]. The NSUN5 demonstrates suppression characteristics in vivo glioma models [58]. NSUN5 gene mutation leads to an un-methylated condition at the C3782 position of 28S rRNA, which leads to a total depletion of protein synthesis and inducing an adaptive translational program under stress collectively [59], as is illustrated that the m^5^C regulators may influence a wide variety of biological functions and metabolism.

In the present study, we applied certain methodological particularities to build a model and evaluated a catalog of genomic characteristics of tumors associated with m^5^C regulators. We obtained a total of 13 m^5^C regulators. The mutations and CNVs of m^5^C regulators are linked to several tumor developments. All cancers carry somatic mutations [60]. UCEC exhibited a significantly higher number of mutations across pan-cancers, analogously TET2, DNMT3B, DNMT3A, and DNMT1 have placed a moderate burden in m^5^C regulator genes. DNMT3B gene mutation was generally higher expression level among various cancers. Here, we sequenced the m^5^C regulator genomes of pan-cancer and providing the first comprehensive remarkable insights into the forces that have shaped various cancer genomes. CNVs play an important role in tumor genesis and progression [61], including amplification and deletion of oncogenes, which may significantly increase the risk of cancer [62]. In this research, the DNMT3B, ALYREF, and NSUN5 showed extensive CNV amplification. In contrast, CNVs such as TET2 and NSUN4 are generally deletion. These results indicated that CNV and the associated gene signatures are useful for early cancer detection and diagnosis, targeted therapeutics, and prediction of prognosis.

The genomic and transcriptomic parameters of various cancers are associated with m^5^C regulators gene expression and activity of the KEGG pathways [63]. We also investigate the gene expression perturbations of m^5^C regulators through 33 cancer types with parallel normal controls. The expression of ALYREF, DNMT1, NSUN2, and TET2 are more positively correlated with the majority of pathways, such as the cell cycle, DNA replication, spliceosome, and nucleotide excision repair pathways. The DNMT1 expression is related to the activation of multiple metabolic pathways, including drug metabolism, lipid metabolism, and nucleic acid biosynthesis signaling pathways. The m^5^C regulators’ pathways are significantly essential for a wide range of biological processes. m5C regulators were also validated involved in malignant activities [64]. Recent studies have demonstrated that the m5C modification in pyruvate kinase muscle isozyme M2 was involved in bladder cancer proliferation and migration. M5C regulator Aly/REF export factor regulated pyruvate kinase muscle isozyme M2 promote the glucose metabolism of bladder cancer [64]. At the same time, the precise molecular modification mechanisms and cellular processes among pan-cancer need further study and deeper exploration for a better prognosis.

For a deeper exploration of the relationship between m^5^C regulators and their clinical outcomes, we describe a comprehensive landscape of m^5^C regulator pathways activities across different cancer types and identify cancer characteristics in relation to clinical outcome. Collectively, we provide robust evidence for the close relationship between cancer-associated clinical relevance and m^5^C regulators. To determine the effect of methylation-based molecular for earlier detection diagnostics in patients with several types of cancer, we systematically analyzed the m^5^C regulators’ pathway activities with the functional and clinically complication for estimating tumor development and progression with potential prognostic value.

## Conclusion

The m5C regulators were differently expressed and showed significantly different CNVs in pan-cancers, which also involved multiple oncogene pathways. In addition, m5C regulators also exhibited prognosis prediction value in pan-cancers. Therefore, our study provides a better understanding of the biology of m5C regulators in pan-cancers, indicating that m5C RNA methylation regulators have the potential to become novel biomarkers and therapeutic targets for various tumors.

## Supplementary Information

**Additional file 1.**

## Data Availability

All of the data involved in this study are available in the public databases which are listed in the “Materials and methods” section.

## Abbreviations

m^5^C: 5-Methylcytosine
TCGA: The Cancer Genome Atlas
CNVs: Copy number variations
m^6^A: N6-methyladenine
m^1^A: N1-methyladenosine
NGS: Next-generation sequencing
FPKM: Fragments per kilobase of transcript per million
DEGs: Differently expression genes
TPM: Transcripts per million
GSVA: Gene set variation analysis
PCC: Pearson correlation coefficient
UCEC: Uterine corpus endometrial carcinoma
KEGG: Kyoto Encyclopedia of Genes and Genomes pathway
PPI: Protein–protein interaction
OS: Overall survival
PFI: Progression-free interval
DSS: Disease-specific survival
DFI: Disease-free interval
ACC: Adrenocortical carcinoma
KICH: Kidney chromophobe
KIRC: Kidney renal clear cell carcinoma
LGG: Brain lower grade glioma
LIHC: Liver hepatocellular carcinoma
HR: Hazard ratio
UVM: Uveal melanoma
CHOL: Cholangiocarcinoma
