# Supplementary figures and images for: Pan-cancer analysis of m5C regulator genes reveals consistent epigenetic landscape changes in multiple cancers
Source: World J Surg Oncol. 2021 Jul 29;19:224. doi: 10.1186/s12957-021-02342-y (PMC8323224; doi:10.1186/s12957-021-02342-y)

category 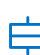 amplification 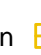 deletion

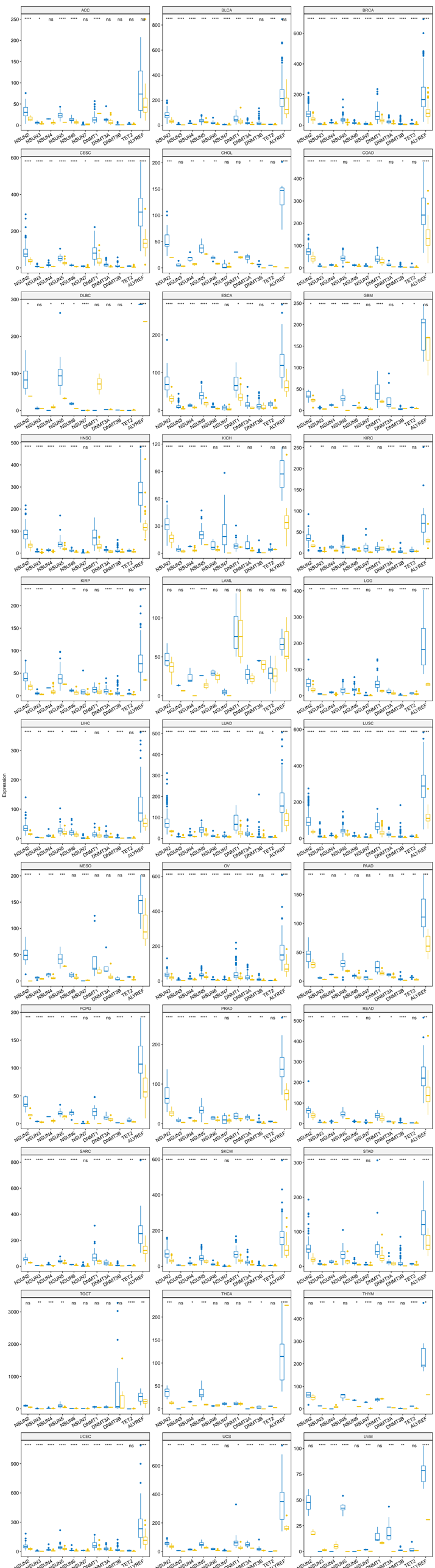

Supplement: Supplementary file 1 — Additional file 1. [file 12957_2021_2342_MOESM1_ESM.pdf]
